# Marginal models for meta-analysis of diagnostic accuracy studies in frequentist and Bayesian framework using rstan and CopulaREMADA

**DOI:** 10.1186/2049-3258-73-S1-O4

**Published:** 2015-09-17

**Authors:** Victoria Nyaga, Aerts Marc, Arbyn Marc

**Affiliations:** 1 Scientifici Institute of Public Health, Brussels, Belgium; 2 University of Hasselt, Diepenbeek, Belgium

## 

Current statistical procedures implemented in general software packages for pooling of diagnostic test accuracy data include hSROC (Rutter and Gatsonis 2001) regression and the bivariate normal model (BRMA) (Reitsma et al. (2005), Arends (2006) and Chu and Cole (2006)). However, these models do not report the overall mean. The hSROC model is less intuitive and therefore less popular while the bivariate normal model has difficulties estimating the correlation parameter when the number studies in the meta-analysis are small and/or when the between-study variances are relatively large. As a result, the between study variance estimates from the BRMA are upwardly biased as they are inflated to compensate for the restriction on correlation parameter (Riley et al. 2007). We present advanced statistical methods for meta-analysis of diagnostic accuracy studies and demonstrate the use of different software packages in R or with an R interface together with code, to apply different model strategies for obtaining appropriate meta-analytic parameter estimates. The focus is on the joint modelling of sensitivity and specificity using copulas as well as using the concept of sharing random components.

The methods are applied to two datasets: the classical example of Glas et al. (2003) on diagnostic accuracy of telomerase as urinary tumour marker for diagnosing primary bladder cancer and a second dataset from a systematic review by Arbyn et al. (2013) that aimed at comparing sensitivity and specificity of human papillomavirus testing versus repeat cytology for triage of minor cytological cervical lesions.

